# Qualitative analysis as a tool for reducing investment risks in post-mining areas located in urban structures

**DOI:** 10.1371/journal.pone.0302058

**Published:** 2024-05-30

**Authors:** Agnieszka Chećko, Zbigniew Jelonek, Iwona Jelonek

**Affiliations:** Institute of Earth Sciences, Faculty of Natural Sciences, University of Silesia in Katowice, Sosnowiec, Poland; Linyi University, CHINA

## Abstract

Urban development is not a process of even and planned progression on residential-industrial sites. Enclaves of high-standard space separate degraded and abandoned areas after industrial use has ended. The idea of the compact city is challenged by the need to search for niches for possible development and even to respond to crisis situations. Changing the approach to postmining sites located inside urban spaces generates an alternative to urban sprawl and the squandering of the stock of fertile suburban agricultural land. The aim of this article is to draw attention to the urban presence of postmining sites, to take a systemic view of ways to identify and describe their specific elements and to determine their impact, from the perspective of different user groups, on the quality of space. This research combined expert knowledge and the practical experience of users to create a model for a multilevel audit of postmining spaces. Knowledge about the postmining environment was transferred to landscape and urban design, creating a universal tool for developing strategies to increase the standard utilitarian functions of revitalized postmining areas. This tool will be useful at an early stage of urban development, management and planning.

## Introduction

**Fig 1:**
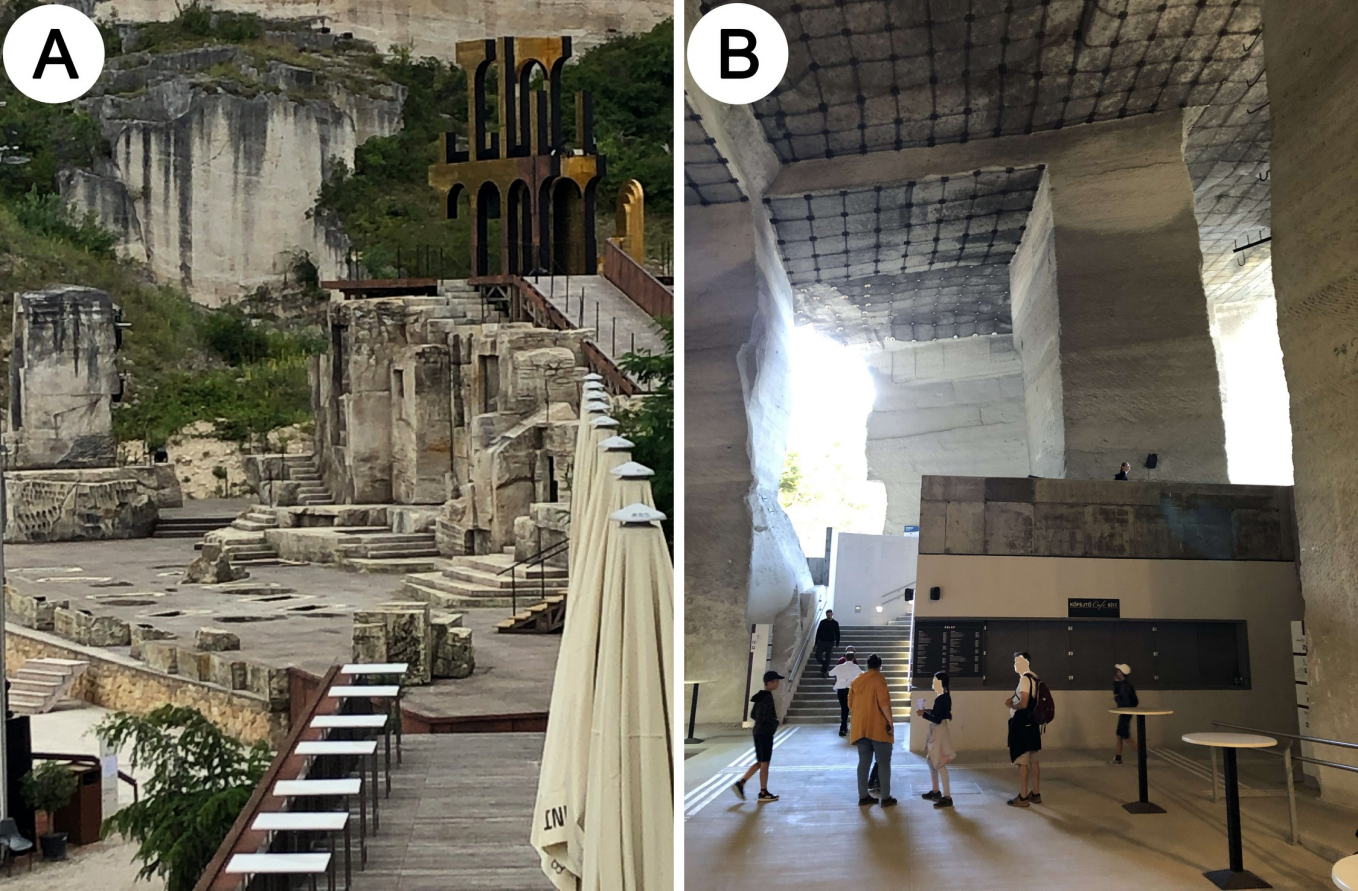
Hungarian and Austrian World Heritage sites. (A) Opera in the Roman quarry in St. Margarethen, Austria, one of the oldest stone quarries in Europe. (B) The Stone Quarry and the Cave Theatre of Fertőrákos, Hungary.

**Fig 2:**
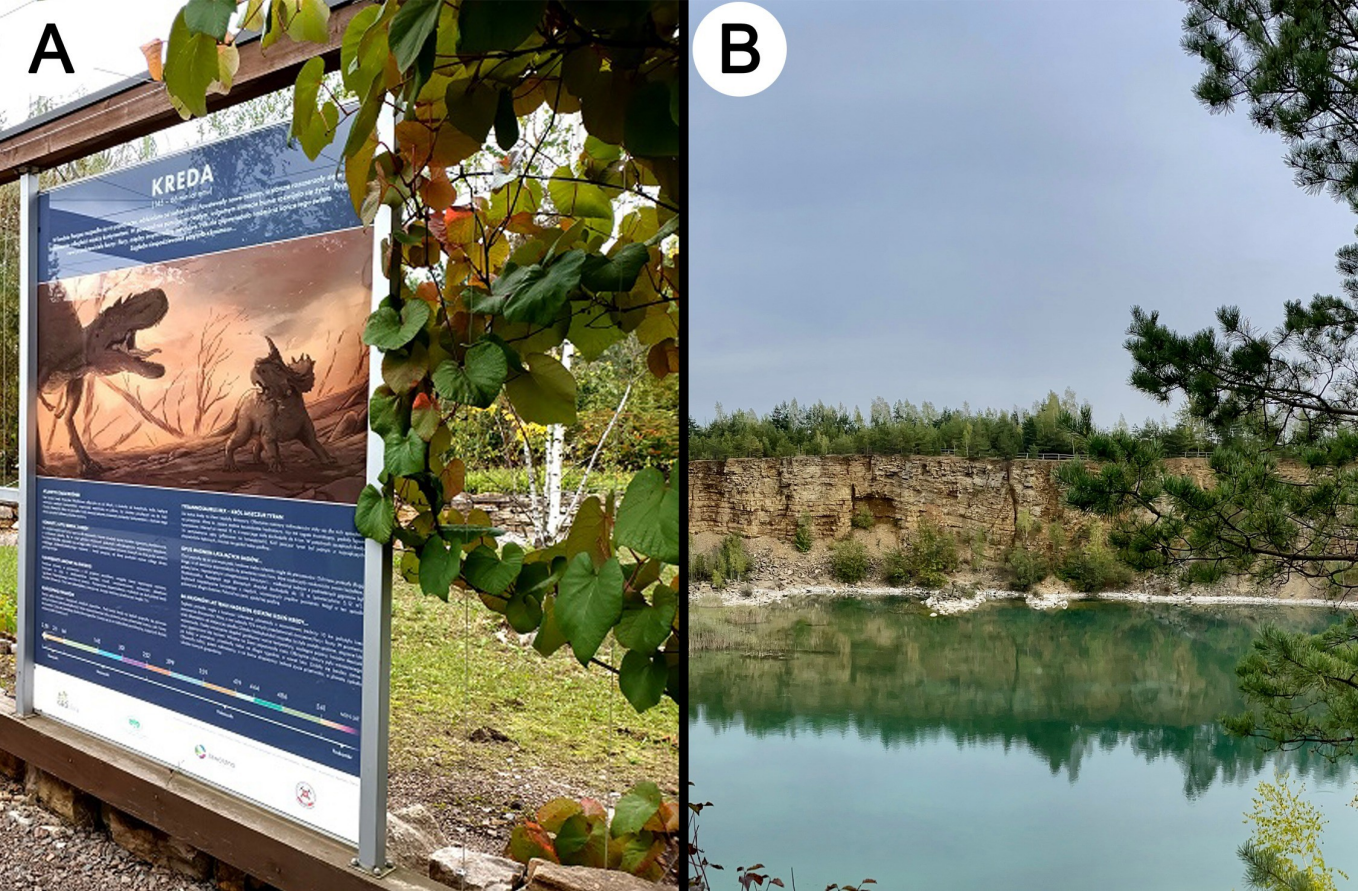
Quarries in Jaworzno, southern Poland. (A) Center for geological education, Sadowa Góra. (B), Polish Maldives, Park Gródek in Jaworzno.

Mineral resources are usually inextricably linked to the development of urban agglomerations. In urban structures or on the periphery of cities, traces of former mining sites are often present. This was mainly for two reasons: first, due to difficulty and cost of transportation, it is more efficient to meet local demands for rock resources with local sources, and second, prosperous mines far from urban centers were ’overgrown’ with urban functions [1]. However, mineral exploitation is always a temporary factor in economic growth. Resource depletion is often associated with a crisis of space in the natural sense [2–5], both socially and economically [6, 7]. Paradoxically, currently, land degraded by mining activities and subsequently abandoned can represent an important reserve of space for agglomerations that lose the opportunity to tap into the resources of green suburbs [8, 9]. The transition from a linear economic model to a closed loop [10] is no longer a matter of choice but rather a necessity and a fact [11]. In the search for directions for the development of postindustrial areas, the stock of mining sites has repeatedly been a resource. The diversity of functions assumed by postmining areas testifies to their high potential for adaptation. The growing number of examples of the use of postmining areas for new functions makes it possible to identify admirable architectural projects. The spaces of former quarries have historical value [12] and can house cultural facilities [13, 14] (Fig 1) and sports stadiums [15], and after they are renaturalized, they can compete with natural areas in attractiveness [16], providing space for nature education and recreation (Fig 2).

There are also mediocre but properly functional spaces that meet the expectations of users; unfortunately, there are also those that function badly and inefficiently or whose adaptation to new functions has led to the devastation of local authenticity and unique values [17]. A quarry is a space separated from its surroundings by mining surfaces [18]. The method of landscape interiors, developed by the Krakow School of Landscape Architecture, was used to establish similarities between the postmining space and the architectural interior [19]. In this method, the description of the space is based on analogies with the architectural interior. The surfaces separating the interior from the surroundings correspond to the walls, floor and vault. This model refers to the components of the quarry without distortion. At the same time, the walls of the excavation and the floor are characterized by universality, legibility and validity, which allows them to be determinants in the description of postmining space.

**Fig 3:**
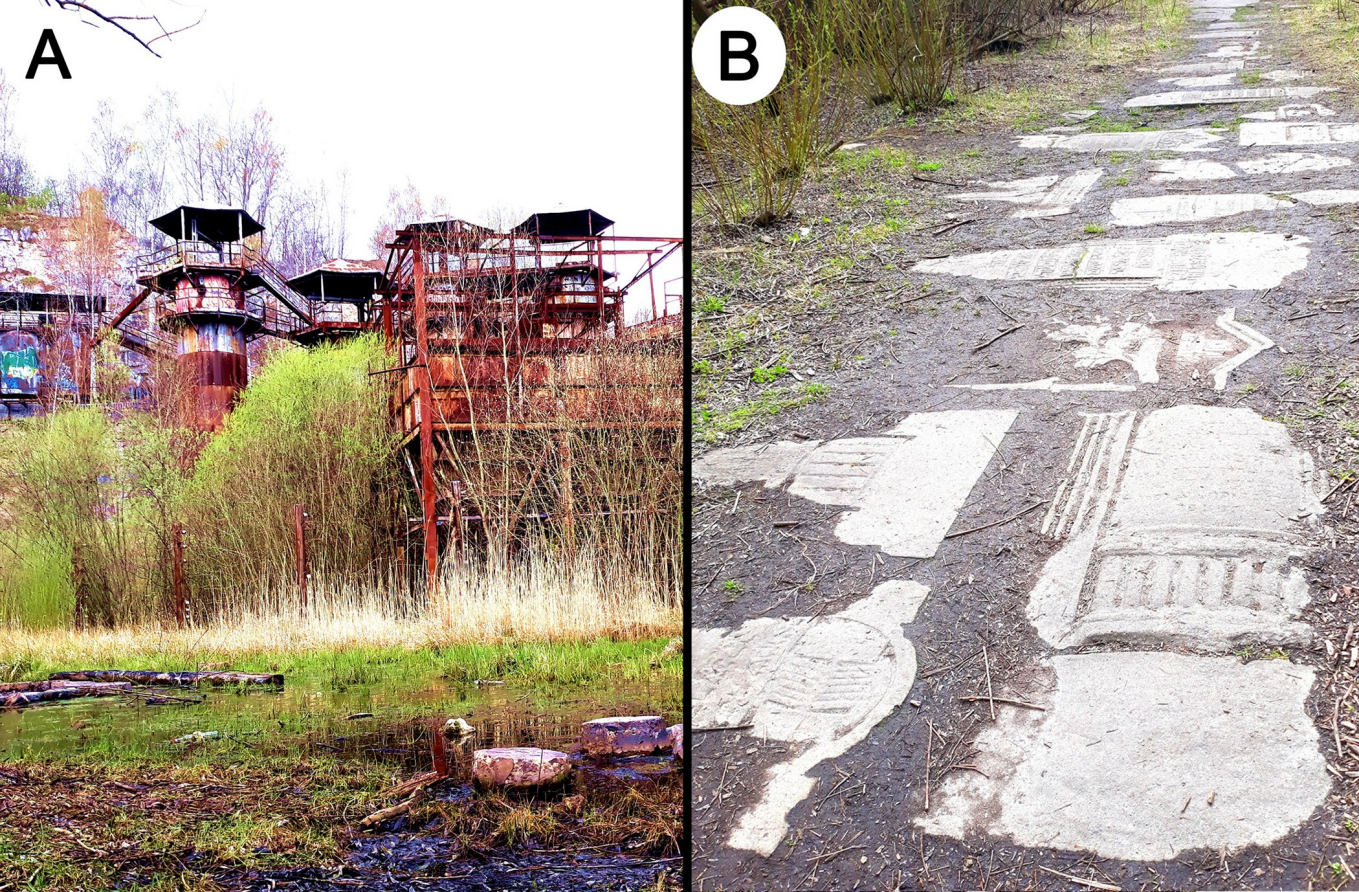
(A) Liban Quarry in Kraków, a place of remembrance for the victims of the Nazi labor camp that operated here during the Kraków WWII occupation. (B) Technical roads paved with tombstones from the Jewish cemetery.

Sick space syndrome results from a disregard or lack of awareness of the complex nature of postmining space [20], which consists of manufactured elements—technical and cultural (Fig 3)—and natural elements—biotic and abiotic [21]. Mistakes made and opportunities exploited can be an important body of knowledge for universal design, provided they are disclosed.

In architecture, a commonly used model for auditing the relationship between a space and its users is the Post Occupancy Evaluation (POE) method [22], which evaluates the state of functionality (in use), based on the experiences of different groups of users of a given space. A new element of this study is the consideration of the cognitive potential of postmining areas in both the cultural and geological heritage spheres. This paper presents a model for the transfer of the assumptions of the qualitative POE method for architectural research to the design of new functions of postoccupancy spaces, focusing on technical, functional and behavioral qualities [23].

### Study area

The main research was carried out in southern Poland, Sadowa Góra in Jaworzno (50.226903746201614, 19.271915340419728) and in Libana in Kraków (50.035278, 19.956944) and was complemented by observations of postmining sites carried out in Austria at The Sankt Margarethen im Burgenland (47°47’56.3"N 16°38’27.4"E) and in Hungary at Fertőrákos (47°43’46.3"N 16°38’43.0"E).

On Polish territory, research was conducted on the border between the Silesian and Lesser Poland agglomerations in the town of Jaworzno, which is an important industrial and technological link between the regions. Since the Middle Ages, zinc and lead ores have been mined here, Poland’s first coal mine (1766) and Poland’s first power station (1898) were established here, and after World War II, large-scale piscina and raw materials for the cement industry were mined here. There are more than 50 sinkholes at the surface level (after coal mining) [24] of various sizes in the city [25]. Today, the city’s industrial character is defined by plans to launch three economic areas with a total area of more than 500 ha. Though the city has an industrial past based on the exploitation and processing of mineral resources, according to the Central Statistical Office, Jaworzno was 5th on the list of the 66 large cities with the greatest proportion of green areas in 2021.

The area that was analyzed, in the main part of the study, is used to mine limestone, a source of raw material for the production of Portland cement. The quarrying industry and the associated cement industry were developed from a small factory founded in 1885 into one of the most modern cement plants in Europe. A period of rapid growth in production was associated with the occupation of more than 100 hectares of meadows and farmland by pits, transport routes and factory buildings at the turn of the 20th century. Development slowed after the end of the Second World War, and a lack of new investment led to the slow degradation of the plant until it was declared bankrupt in 1995 and the fields were abandoned without being rehabilitated. The most visible traces of the cement factory’s activities are the five pits that serve various formal and informal functions. These include a geological education park with an area of 8 ha (50°13’36.6 "N 19°16’20.5 "E), tanks for divers with an area of 15 ha (50°13’41.6 "N 19°18’42.1 "E), and an arboretum established on a municipal waste dump with an area of 17 ha (50°13’39.4 "N 19°19’17.2 "E). There is also an undeveloped excavation pit with an area of 14 ha (50°13’46.7 "N 19°16’51.2 "E), which contains an abandoned underground storage facility for explosives used in the mining process.

The close proximity of buildings with similar technical parameters but different functions allowed the analysis of a wide range of relationships between users and space features in different functional contexts.

This research was complemented by observations at quarries with similar spatial and technical conditions, combining elements of natural and cultural heritage used as public spaces: the Liban Quarry in Kraków, Poland, with an area of 18 ha (50°03’71.2 "N, 19°95’86.1 "E), that is used as place of remembrance for victims of the Nazi labor camp that operated here during the WWII occupation [26]; the Sankt Margarethen im Burgenland, Austria (47°80’36.10 "N, 16°63’43.3 "E), which houses an opera in a 35 ha quarry [27]; and the Fertőrákos Kőfejtő cave theatre near Solon, Hungary (47°72’67.6"N, 16°64’46.9"E), with an area of 10 ha.

## Methods

This study, based on the assumptions of the POE methodology developed by the team of Preiser, Rabinowitz and White, was used to assess the fit between space and user needs. The methodology, however, required adaptation to the specific conditions of the postuse space. Adapting the method required changes to the qualitative categories and to the techniques and tools selected to meet the needs of a space combining both manufactured and natural elements. For the purposes of the research, simple factor-effect analyses were used, similar to experiments carried out in both the natural sciences and in fields of the humanities related to the space of human life. The research consisted of expert and participatory parts.

The exploratory part consisted of selecting the key features of the objects produced or revealed in the exploitation process and describing the site. The selection of determinants was based on a literature review, archival data, and study visits. The expert phase combined the perspective of the designer, supported by the opinions of experts from the earth sciences, publicly available spatial data and archival data resources. The expert analysis comprised field surveys and the acquisition of data from current GIS (*geographical information system*) spatial information sources and historical sources collected in geological and military archives (Map Archive of the Military Geographical Institute 1919–1939.) Reference to historical sources was used to identify objects created in the process of exploiting mineral resources, especially those whose morphology was highly distorted in the landscape after long-term abandonment.

The analysis of object morphology focused on geotechnical and hydrogeological considerations. The purpose of the geotechnical investigations was to identify the features of the site related to resistance to external factors in the context of development. Hydrogeological studies determined the qualitative and quantitative status of water resource availability. The study was followed by an inventory of technical and natural facilities. The key features of the space in question defined in the expert phase were used to develop scenarios for participatory research, which was carried out to establish the relationships of the individual features with the users.

The main part of the study consisted of participatory research: focus meetings, face-to-face interviews including an interview with a person with blindness and a person in a wheelchair, direct observations, analysis of data from video surveillance recordings, questionnaire surveys, and observations of use footprints. Two stakeholder groups participated in the focus meetings.

Group I consisted of administrators of postmining facilities that were adapted to new functions (7). Focus group II consisted of families with children (10) and senior citizens (4).

Participants were selected to match the target market, the structure of which was established based on survey data from a group of 209 participants.

The size of the group allowed each participant to speak and share his or her experiences and ensured interaction between participants. The diverse composition of the groups was dictated by the desire to obtain answers formulated from different perspectives, arising from different professional and personal experiences.

The research was overseen by a facilitator, who ensured that the research followed the script, directing the conversation toward relevant topics, stimulating new ideas and taking responsibility for the smooth operation of the research. The development of scenarios for the focus groups ensured that all questions of interest were answered and that an orderly analogy was maintained for all groups. At the same time, the moderator was tasked with capturing and using nonverbal signals to communicate participants’ attitudes on the issue at hand and with including in the discussion any directions that were not taken into account when developing the scenarios. A person was also appointed to be responsible for the annotation of day-to-day meetings. The focus group methodology was sufficient to draw conclusions for the qualitative research, but these findings were supplemented by interviews at the pilot site with 106 participants, as well as field observations and a trace study and analysis of the video surveillance recordings, drawn from 17 installed cameras. The above research tools were then used to develop as simple a model as possible for the sake of the versatility of the tool and the possibility of modifying it according to the specifics of the postmining site. In the end, using the POE method to organize the research results, an evaluation matrix was created according to the principle of qualitative categorization adapted to the conditions of the postmining space, covering the areas of technical, organizational, behavioral and cognitive qualities.

## Results and discussion

In the expert phase, determinants were selected for the description of the objects under study, their relationships with users were analyzed, and the quality areas for which they were relevant were identified, thus defining their categories (Table 1).

**Table 1:** Analysis of the impact of the postexploitation spatial elements on the categories and spatial quality in the study area: T—technical, O—organizational, B—behavioral, P–cognitive.

Determinant	Scope of impact	Scope of intervention	Impact on the quality area			
			T	O	B	P
Geometry	Communicating conditions related to the effort of overcoming the height difference	Designation of accessibility sites (lowering of land)				
		Introducing technical adaptations for accessibility (stairs, ramps, lifts)				
	Conditions for practicing terrain Elevation sports	Designation of safe challenge sites (possibility to anchor belays for climbing)				
	Exposure conditions	Use of natural wall lighting to display geological structures and objects				
		Introduction of night-time lighting				
	Range of visibility outside the quarry and inside the quarry	Designation of sites with an attractive range of visibility				
		Introduction of exposure explanations				
		Preparation of technical structures to make observation more attractive (bridges, platforms, observation towers)				
		Optical equipment				
	Conditions for pond access	Designation of sites for nature observation				
		Designation of beaches in areas with gentle slopes				
		Shaping a gentle descent into the reservoir				
		Designation of viewpoints in cliff areas				
		Marking of danger zones (difficult access, sudden change in depth, significant temperature differences)				
	Conditions for the movement of air masses	Use of natural barriers (tall vegetation, rock features)				
		Introduction of technical barriers (sails, partitions, canopies)				
	Acoustic conditions	Use of natural barriers to dampen external noise				
		Introduction of technical shutters to dampen external noise				
		Use of quarry walls as an element of sound amplification				
Spatial orientation	Layer gradient conditions	The fall of layers toward the excavation increases the intensity of erosion processes resulting in an increased risk of rock fall.				
	Sunshine conditions (light and shade zones)	Use of natural obscurants to limit sunlight access (tall vegetation, rocks)				
		Introduction of technical barriers (sails, partitions, canopies)				
	Determination of the conditions for the movement of air	Use of natural barriers to protect against wind (tall vegetation, rock features)				
		Introduction of technical barriers (sails, partitions, canopies)				
Structural conditions	Identification of safety conditions associated with rockfall	Exclusion of hazardous areas from use				
		Introduction of technical safeguards (nets, anchors)				
	Foundation conditions on the quarry floor	Taking into account foundation conditions in development planning				
	Trench conditions	Consideration of excavation difficulties for linear installations and underground structures				
	Conditions of water supply and drainage	Consideration of rainwater capture in water-deficient areas				
		Consideration of drainage in drainless areas				
	Conditions for green cover, in particular the possibility of tree planting	Consideration of the need for soil restoration in areas devoid of vegetation				
		Species composition analysis with recommendation for tall vegetation with shallow root system				
Water conditions	Specification of the possibility of obtaining water for green maintenance and technical purposes	Identification of options for organizing underground intakes				
		Consideration of the need to quench water for plant watering to avoid thermal shock				
	Identification of the feasibility of surface water reservoirs	Organization of park spaces (without swimming options)				
		Swimming arrangements				
		Organization of water sports and recreation sites				
Geochemical conditions	Species composition of natural vegetation and in plant collections	Vegetation selection dependent on natural soil composition				
		Selection of vegetation depending on the contaminants present in the ground				
	Definition of the scope of application of water	Identifying opportunities for water use				
		Determination of water destination				
Geostations	Specification of the possibility of making space available	Determination of the importance of the position in order to establish the function (scientific, educational, cognitive)				
		Preparation of the exposition by including it in the traffic routes, providing a description, introducing night lighting				
	Identification of collisions with other forms of development	Collision with vegetation				
		Collision with cultural elements				
Cultural facilities	Determination of the possibility of introducing cognitive functions	Determining the importance of the position in order to establish the function (scientific, educational, cognitive)				
	Determination of how to protect and conserve historic and cultural sites	Integration of the facility into the traffic route, labeling, introduction of night-time lighting				

The prerequisites for a feature to be accepted as a key feature were a significant impact on the user (magnitude of impact), occurrence in every facility (universality), and an obvious link to functionality (readability).

The elements of pit geometry (height and width), spatial orientation, geochemical properties, rock mass structure, water conditions, and natural and cultural heritage elements were identified as determinants.

The role of individual elements was determined by the opinions of end users and facility administrators.

A key element of the physical accessibility of the site is the connection to the outside environment (at the pit boundary). In the case of accessible excavations, the height difference between the lower and upper edges of the quarry wall is an important factor. In all the facilities analyzed, the zones with the lowest relative heights were the most predisposed for use as an entrance–area—they required the least effort to overcome the height difference. This factor was particularly important for people with special needs, e.g., elderly people, people with mobility constraints, especially wheelchair users and families with children in strollers.

The difference in height between the interior of the quarry and its surroundings in all the facilities studied required technical support. The facilities included ramps and stairs equipped with handrails. Respondents with special needs pointed out that the slope of the ramps should be chosen to ensure that the energy expenditure for traveling up the ramp and braking the wheelchairs on descent is optimized. Fit people preferred to use the stairs because they reduced the time needed to cover the up-and-down distance. These opinions were confirmed by footprint observations, where only ramps were in operation and trampled ’shortcuts’ were visible. A combination of both solutions was optimal.

In the administrators’ group, attention was drawn to the need to reserve a sufficiently large area for the organization of long rallies, occupying a significant space.

The internal communication routes serve to organize traffic at the bottom of the quarry, and their role is to lead to points of key importance for the functioning of the site (points that are culturally or naturally attractive), integrating the site with places of importance for rest, shelter from the weather, supply or sanitation. The respondents expected varied walking routes and a levelled and paved surface. Those taking part in the study with visual deficits (blind and visually impaired) pointed out that the surface of small stones produces sounds that provide information about the movement of others in the surroundings.

The use of loose material paving allowed the geological structures on the quarry floor to be preserved (which was important in the case of the Orchard Mountain site). Those taking part in the survey were aware of this and, where improvements to the standard conflicted with the preservation of sites of natural value, participants were prepared to use less comfortable solutions but stressed that the reason for the lower standard of paving should be clearly explained.

**Fig 4:**
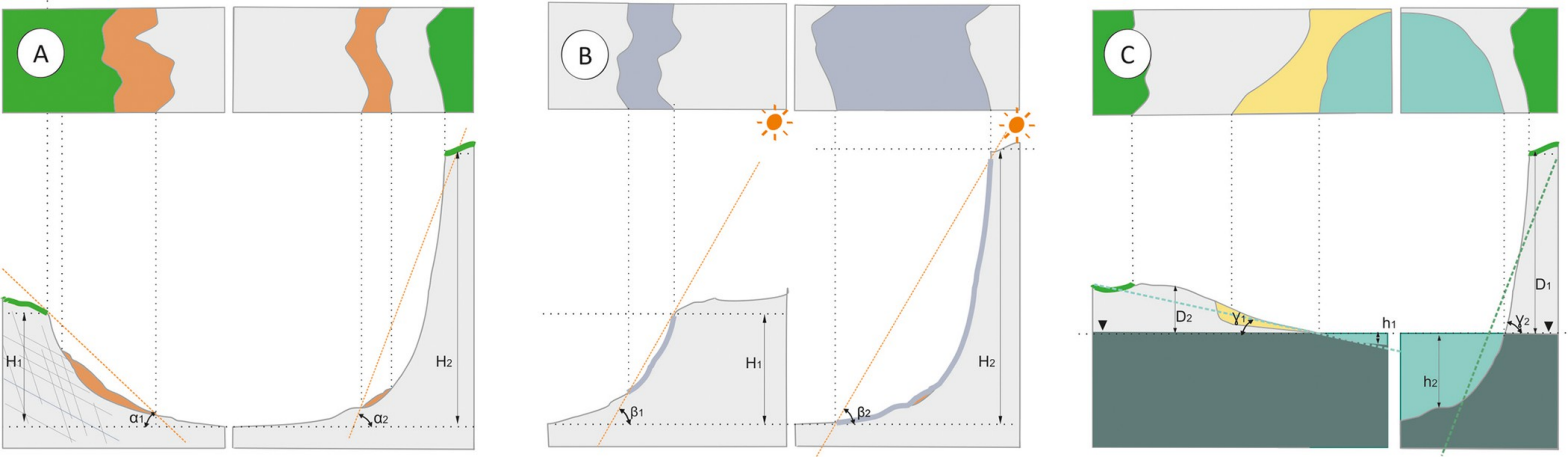
The geometry of quarry walls in the context of land use. (A) Rock resistance versus structural ground conditions. (B) Slope height vs. sunlight conditions. (C) Slope vs. conditions for making water bodies available for recreation.

According to the respondents, important issues for trails in open areas were sunshine and protection from the wind and rain. According to those who visited in person, these were major hindrances to using the facility in autumn and winter. These opinions were confirmed by the video surveillance records. The mellowing slopes allowed the land to be used for informal descents, motocross rides and the organization of extreme bike descents (Fig 4A). On the other hand, observations of the footprints left behind showed that, in the absence of cover, users descended from the designated trails, searching for a possible route in shadow zones (Fig 4B), under trees or along walls. However, the presence of surface water required safety precautions for users, with dangers mainly posed by steep banks (Fig 4C) and, in winter, the desire to use the surface of the reservoir as an ice rink. Video surveillance records also confirmed users’ entries on the frozen surface of the reservoir.

**Fig 5:**
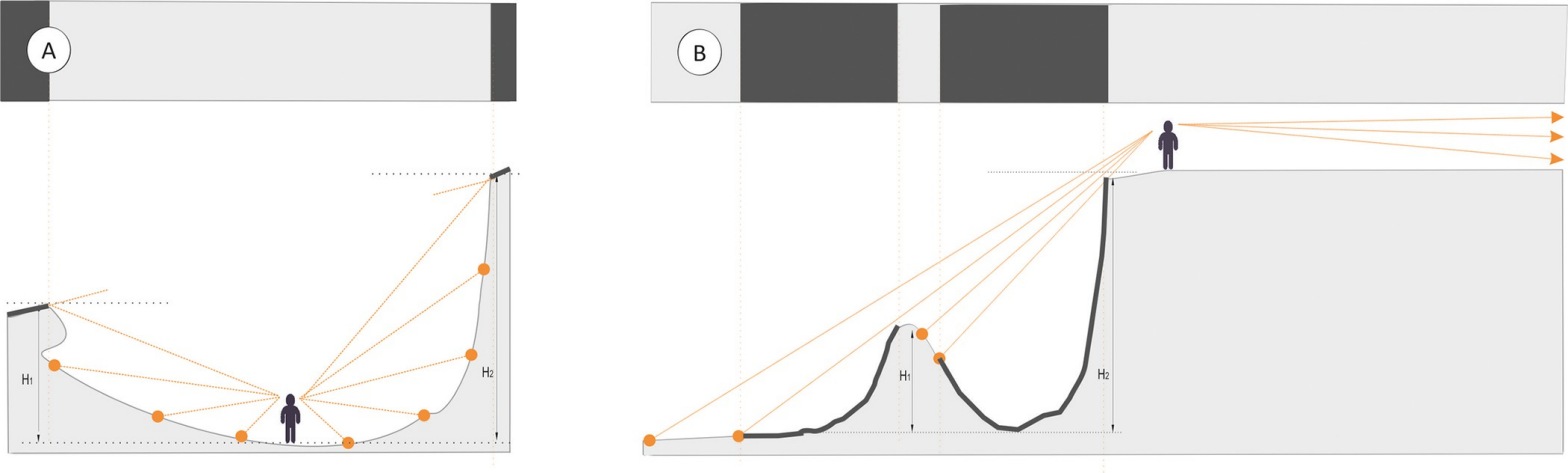
The range of visibility depends on the location of the observation point. (A) An observer at the bottom of the quarry. (B) An observer at the top of the quarry.

For the focus group participants, the danger of rockfalls was not obvious. In contrast, walks along the lower edge, despite the shortened perspective (Fig 5A), were attractive because of the satisfaction of cognitive needs (observation, a search for fossils and minerals) and the possibility of using walls as a background for photography.

Administrators noted the difficulty of providing sufficient shade due to the difficult rooting conditions for tall vegetation; however, the creation of various types of weather screens was associated with incidents of vandalism. Site lighting was used as a preventive measure. However, the administrators noted that the organization of lighting was associated with a technical difficulty when digging trenches for the electrical cables, a difficulty that had not been foreseen at the design stage.

In the case of the pits, trails needed to be maintained both at the bottom of the quarry and along the upper edges. These trails mainly have a viewing and leading function. The advantage of walking trails at elevation (Fig 5B) is that the perspective can be changed.

Survey participants attributed high attractiveness to the highest point of the surroundings, while they also expected information about the effort required to reach high points and what they could expect when they reached it. The respondents also expected resting places and elements that increase the attractiveness of the view, such as viewing platforms or observation towers and optical devices, to support the observations. Walking along the edges of the walls generally did not make users feel unsafe or at risk of falling. Observations of footprints confirmed instances of departing the footpaths and traces of camping at the edge of the excavation.

Administrators confirmed the attractiveness of heights but were aware of the need for physical and preventive measures to protect against falls. During the focus group meetings, incidents of falls from high points, including fatalities, were confirmed. Administrators struggled with a lack of information on effective methods to counteract these behaviors and were unable to mention good practices or standards.

The role of footpaths is to connect important points on the site with each other and with the entrance-exit zone, acting as nodes in the traffic network. The points that users aim at are objects (points, lines, areas) with increased attractiveness. This study mainly considered the natural elements revealed by mining and the equipment and structures associated with mining activities as elements that determine the identity of a place.

The space created in the course of excavation is a result of the technological process, but its frame remains the natural formations, and it is these elements that determine its identity. The surfaces of the pit walls and bottoms are the most exposed elements of the postmining landscape. In the focus survey, users assessed the attractiveness of the exposures mainly in terms of aesthetics. Those who joined the survey were not able to determine the educational and scientific significance of the site; during the meeting, they were provided with explanations regarding the genesis of the geological formations exposed in the survey area, which resulted in a change in their assessment of the attractiveness of the site and in assigning a higher rating to the educational value. The relationship of the value of biological to geological objects also changed in their assessment. At the same time, the study participants declared an expectation that information about the genesis of the site would be placed in the field.

From the administrators’ perspective, the scientific and educational values were a major element in determining the attractiveness of the site. However, at the design stage, there was no inventory or valorization of the sites necessary for preservation, conservation or restoration. During the course of the investment tasks, there was a need to change the location of the facilities, which, at the stage of preparing the project site, involved revealing valuable structures. The greatest problem was the exposure of horizontal planes in the subsoil, and maintenance was a problem due to exposure to weathering (freezing). It was also reported that there was a problem with installing technical installations and structures to protect the uninventoried extent of protected structures. The exposure of vertical walls, according to the administrators’ declarations, was easier to protect, although the preparation of descriptions on information boards had to be designed to prevent obscuring the presented object; the most serious difficulty was the danger of falling rock fragments.

In technical terms, resistant walls could be used for anchoring structural elements and protection systems, e.g., for climbing. At the same time, they were a major impediment to the execution of construction, which was important for building retention basins or trenches for underground installations (electrical, water supply or sewage systems). The trenches were also important for aesthetic reasons and because in a climate where subzero temperatures are recorded, water in pipelines at the surface level can freeze and, consequently, lead to bursting.

In the case of rocks with a low resistance to weathering, there was weathering of the walls and accumulation at the foot of the mounds, and the accumulated material worsened the exposure conditions but facilitated habitat establishment. The material could also be used in landscaping.

The problem for administrators was reconciling the co-occurrence of biotic and abiotic objects and the selection of plants. Introducing plants into former mining areas allowed for an increase in biodiversity [28], improved the aesthetics of the site and enhanced the comfort of users. The presence of greenery helps to lower the temperature during hot weather, increase the humidity of the air and reduce the force of the wind. One difficulty in designing the greenery was the lack of soil. In the study area, the administrators established plant collections in specially prepared plots, to which a soil mixture corresponding to the mix in the immediate surroundings was provided. The solution considered the selection of plants suited to the pH and mineral composition of the soil, and large fluctuations in temperature and humidity. A groundwater intake was constructed for the water supply. The solution did not take into account the susceptibility of the elevated plots to frost and too low a groundwater temperature, which was poorly tolerated by some species at high air temperatures. During the operation of the facility, a retention basin was constructed for water heating, which was also used as a recreational basin.

Water conditions were a feature of great importance to users. The possibility of accessing a body of water was associated with recreation and the possibility of observing aquatic biocenoses was also important because of better thermal comfort. Users did not associate any negative associations with the presence of water. For the administrators, this factor was also very important, as access to surface water increased the attractiveness of the facility and provided the possibility of using the water for green area maintenance.

Historic mining sites are highly valuable for quarrying and using stone. They are also often the scene of tragic events related to the organization of forced labor. Administrators used elements of mining and geological heritages as important elements in the construction of the identity of a place. However, these objects require measures to protect them, to explain their significance, to expose them properly and to eliminate the factors limiting these activities. Elements produced during the exploitation process, such as buildings or equipment related to mining and processing the raw material, were assessed by the respondents as interesting and visually attractive. The respondents declared a narrow knowledge of topics related to mineral exploitation, but they felt the need to acquire this knowledge.

From the perspective of the administrators, who share a sense of the high attractiveness of the sites, the difficulty in making the sites accessible was grounded in ensuring safety considerations and the lack of substantive knowledge on conservation activities.

**Fig 6:**
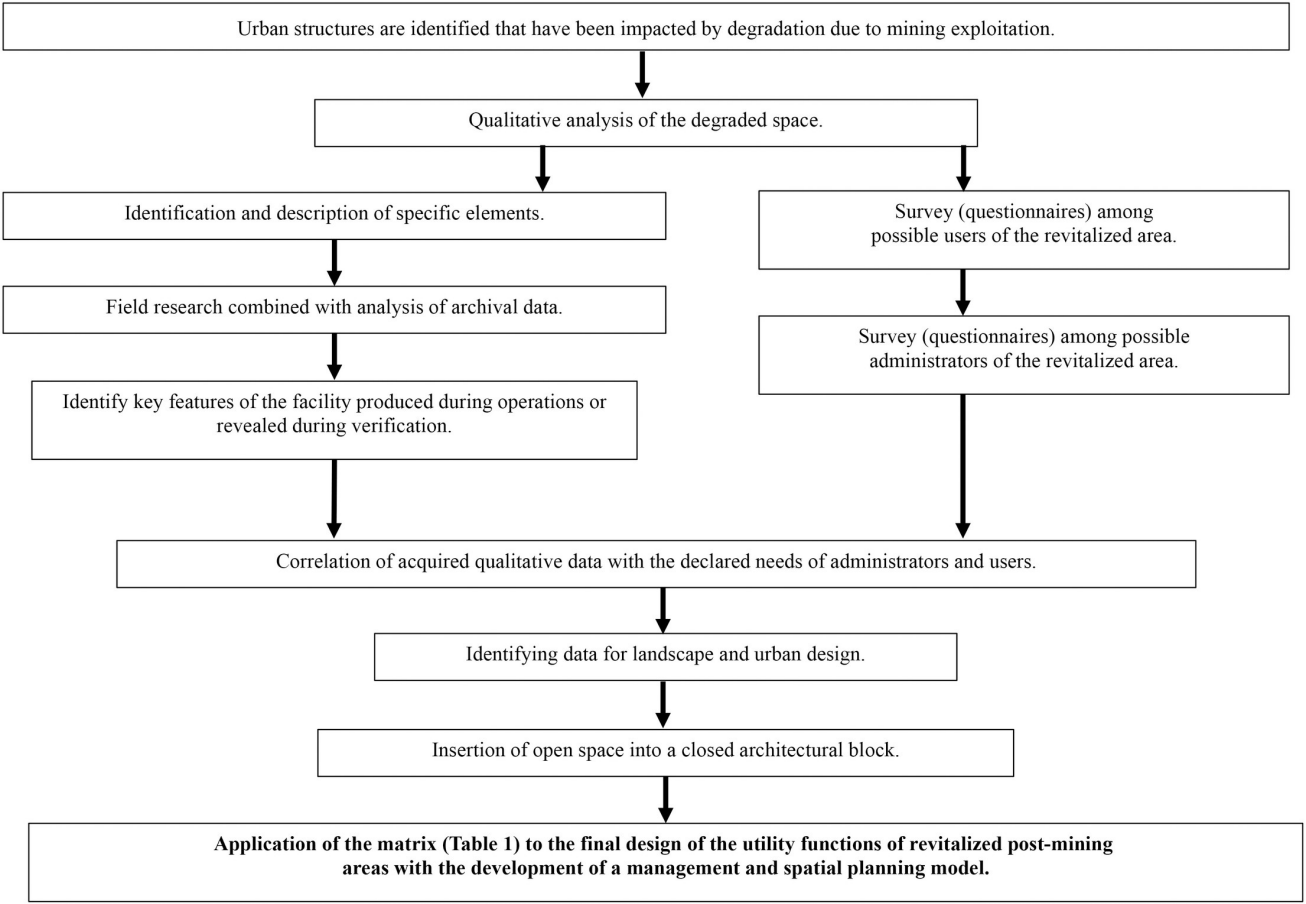
Flowchart of a new method for designing postmining spaces embedded in urban areas.

During the focus groups, it was confirmed that the key features of the quarry space identified in the expert phase, such as spatial dimensions and orientation, structure and chemical composition, availability of water resources, and natural, technical and historical elements of the geographic identity, directly influence the different user groups and are, therefore, determinants of the quality of the space. Each of the listed characteristics was assigned significance in the creation of a quality category, according to the POE methodology (subject to modification for postmining sites), creating a qualitative assessment scheme (Fig 6).

## Conclusions

The development of cities in the postindustrial era depends on the ability to create a loop in which the degradation of the quality of the space, due to industrial activity, does not close off the possibility of reusing it in a new, different function. Surface mining of minerals is a type of use of environmental resources that always leads to significant, irreversible changes to space. Both the scale of environmental change caused by mining activities and the dynamics of environmental change make it impossible for postmining areas to return to their original functions and necessitate the identification of new development models for them. This paper describes the process of identifying the basic conditions and quality of postmining space using the example of abandoned quarries following the exploitation of rock resources in urban structures. The practice of repurposing postmining sites located in southern Europe confirms that these sites can be important resources responding to urban space deficits. The diversity of parameters characterizing postmining space makes it difficult to model the sequence of activities leading to the creation of a functional program for restoring new public utilities in historic mining sites. Individual examples of the adaptation of quarries for social spaces, although outstanding, are of such a unique nature that they do not allow designers and owners to adapt individual aspects directly from them. This study focuses on the most basic, but always present, elements of space in mining pits in terms of their impact on the user, such as geometry, spatial orientation or rock mass structure, as well as on features that are key to development. These features both limit and enhance utility functions, such as the availability of water and the presence of unique natural (mainly geological) and cultural objects. Methods used in urban planning were applied, implementing a new development perspective on the space created by open-pit mining. The proposed process resulting from this research uses elements of technical and natural sciences and environmental psychology. The use of uncomplicated analyses in a logical sequence and an interdisciplinary approach created a versatile tool that is useful for different types of postmining sites. The model makes it possible to identify areas of impact on the user and to identify courses of action to improve the quality of the postmining space and thus to reduce investment risks. The practical recommendations and guidelines for the model presented above were tested in an abandoned pit resulting from the exploitation of carbonate rock. This model can support urban development in the spirit of a closed-loop economy, enriching the management strategies for postmining areas altered by open-cast mining methods during the extraction of rock materials.

## Supporting information

S1 File Summary of respondents’ answers. (XLSX)
